# Sustained Antifungal Protection of Peanuts Using Encapsulated Essential Oils

**DOI:** 10.3390/molecules31010038

**Published:** 2025-12-22

**Authors:** Narjisse Mokhtari, Hammadi El Farissi, Francesco Cacciola, Yousra Mdarhri, Abderrahman Bouassab, Mohamed Chabbi

**Affiliations:** 1 Laboratory of Physical Chemistry of Materials, Natural Substances and Environment, Faculty of Sciences and Technologies of Tangier, Abdelmalek Essaadi University, Tanger 90010, Morocco; y.mdarhir@etu.uae.ac.ma (Y.M.); a.bouassab@uae.ac.ma (A.B.); m.chabbi@uae.ac.ma (M.C.); 2 Chemical Engineering for Resources Valorization Group (UAE/L01FST), Faculty of Science and Technology, Abdelmalek Essaadi University, Tangier 90010, Morocco; 3 Messina Institute of Technology c/o Department of Chemical, Biological, Pharmaceutical and Environmental Sciences, Former Veterinary School, University of Messina, Viale G. Palatucci snc, 98168 Messina, Italy

**Keywords:** fungal reduction, *Arachis hypogaea*, *Origanum compactum*, *Myrtus communis*, microencapsulation, *Aspergillus flavus*, germination power

## Abstract

Essential oils (EOs) are promising bio-preservatives for oilseeds; however, their high volatility and strong aroma limit practical applications. In this study, we developed a dual-size microencapsulated formulation of oregano (*Origanum compactum*) and myrtle (*Myrthus communis*) EOs (75:25, *w*/*w*) using gelatin–gum arabic complex coacervation, and evaluated its antifungal efficacy and effect on seed viability in peanuts. GC-MS analysis of the EO blend identified carvacrol (33.83%) as the dominant constituent. The microcapsules exhibited an encapsulation efficiency of 83.56% and were produced in a 70% small/30% large particle ratio to ensure both immediate and sustained vapor release. In vapor-phase assays against toxigenic *A. flavus* (RP-6), both free and encapsulated EOs inhibited fungal growth in a dose-dependent manner and achieved complete suppression at concentrations ≥0.2 µL mL^−1^, whereas the wall material alone showed no activity. In a 120-day microcosm storage experiment (0.2 mg EO g^−1^ kernels; 0.96 mg microcapsules g^−1^), treated peanuts showed an immediate reduction in total fungal load from 3.52 to 1.48 log_10_ CFU g^−1^ (≈58%), which stabilized near 1.42–1.43 log_10_ CFU g^−1^ up to 90 days, while the control samples increased to 4.25 log_10_ CFU g^−1^ by day 120. The formulation effectively suppressed major storage fungi, including *Aspergillus* sections *Flavi* and *Nigri*, *Penicillium* spp., *Rhizopus*, *Fusarium*, and *Alternaria*. The antioxidant activity (DPPH assay) was retained after encapsulation (IC_50_: 0.52 mg mL^−1^ encapsulated vs. 0.58 mg mL^−1^ free). Germination power remained comparable to the control throughout storage (≈50–52%), indicating no adverse impact on seed viability. These findings demonstrate that vapor-active, dual-size microencapsulation of oregano-myrtle EOs offers a practical and sustainable approach to enhance peanut safety during storage without compromising germination potential.

## 1. Introduction

Peanuts (*Arachis hypogaea* L.) are among the most widely cultivated legume crops worldwide, valued for both their high nutritional quality and economic importance. They are rich in high-quality proteins, unsaturated fatty acids, dietary fiber, potassium, magnesium, and B vitamins, making them a major component of human diets and agro-industrial systems [1,2]. However, peanuts remain highly vulnerable to fungal contamination during postharvest storage, particularly by species belonging to the genera *Aspergillus* and *Penicillium*, which can severely compromise both product quality and safety. Among these, *Aspergillus flavus* is a filamentous fungus of major concern due to its role in food spoilage and its capacity to produce aflatoxins, making it a relevant target organism for evaluating antifungal or inhibitory strategies. Beyond economic losses, fungal growth poses serious public health risks due to the production of mycotoxins, especially aflatoxins, which are among the most potent naturally occurring hepatocarcinogens [3,4].

In parallel with food safety concerns, preserving the germination capacity of peanuts intended for planting is essential for agricultural sustainability and long-term crop productivity [5]. Although conventional chemical fungicides are effective, their extensive use raises increasing concerns related to environmental persistence, non-target toxicity, and human exposure. This context strongly reinforces the need for safer, bio-based preservation strategies that are compatible with both food protection and seed viability.

Microencapsulation has emerged as a powerful technological approach to improve the stability, controlled release, and biological efficacy of natural bioactive compounds used in food preservation and crop protection. For essential oils (EOs), encapsulation effectively reduces their volatility, and intense aroma can be attenuated while preserving sustained vapor phase [6,7]. In this context, green dual-size microencapsulation of *Origanum compactum* (*O. compactum*) and *Myrtus communis* (*M. communis*) essential oils represents an innovative approach for achieving prolonged release, enhanced stability, and improved antifungal efficiency in stored peanuts [4]. These two plant-derived oils were selected for their complementary chemical profiles: *O. compactum* is rich in phenolic monoterpenes such as carvacrol and thymol, which exhibit strong antifungal activity against aflatoxigenic *Aspergillus* and storage-associated *Penicillium* species [8,9], whereas *M. communis* is dominated by oxygenated monoterpenes, notably 1,8-cineole and α-pinene, which provide broader but milder antifungal effects, lower volatility, and a more acceptable sensory profile [10,11]. Their combination at a 75:25 oregano-to-myrtle ratio was therefore rationally designed to promote synergistic interactions between phenolic and oxygenated compounds, ensuring an optimal balance between antifungal potency, vapor-phase persistence, formulation stability, and practical applicability in peanut storage systems.

Building on these considerations, the present study evaluates the antifungal performance of a dual-size microencapsulated formulation containing *O. compactum* and *M. communis* essential oils against storage-associated fungi in in-shell peanuts, while simultaneously assessing its impact on seed germination to ensure preservation of planting quality. By combining in vitro vapor-phase antifungal assays with long-term microcosm storage experiments, the work aims to develop a practical, environmentally friendly strategy to improve peanut safety and quality during storage.

To the best of our knowledge, this is the first report describing the use of a dual-size microencapsulation system based on oregano-myrtle essential oils for sustained antifungal protection of peanuts. This innovative design enables controlled vapor release over time, ensuring effective fungal suppression without compromising seed viability. The findings support the development of green preservation technologies for oilseeds and contribute to integrated approaches that align food safety and seed quality management.

## 2. Results and Discussion

### 2.1. Chemical Characterization of the EO Blend

The essential oil mixture was successfully encapsulated, achieving an encapsulation efficiency of 83.56%. Table 1 summarizes the compositional profile compared to the individual EOs. Across all samples, 44 compounds were identified. In the blended oil, Carvacrol appeared as the dominant constituent, 33.83%, although its proportion in pure *O. compactum* EO was only 3.07% and it was absent in *M. communis*. Since this value does not follow proportional mass balance expectations, it cannot be interpreted as evidence of a chemical or biosynthetic conversion during blending. Instead, the unexpectedly high carvacrol percentage must be viewed as an analytical outcome of GC–MS behavior in mixed matrices, a phenomenon previously noted in studies examining compositional shifts and interactions in essential oil mixtures [12].

Several factors may contribute to this apparent elevation. First, matrix effects may alter detector response and peak intensity when phenolic monoterpenes are present in complex mixtures. Second, co-elution or overlap of aromatic compounds near the carvacrol retention time can inflate the integrated peak area. Third, baseline interactions resulting from more abundant aromatics, such as m-cymene (13.40% in the blend), may affect peak quantification. These explanations are more consistent with known analytical behavior than invoking in planta biosynthetic pathways. The biogenetic formation of thymol and carvacrol from γ-terpinene is well described in living plant tissues and involves terpene synthases, cytochrome P450 monooxygenases, and short-chain dehydrogenases/reductases [13]. Such enzyme-mediated reactions cannot occur in distilled essential oils under the conditions of the present study. For this reason, a moderate decrease in γ-terpinene (from 28.35% to 17.41%) is not interpreted as chemical conversion.

The non-detection of eucalyptol, the principal compound of pure *M. communis* EO (65.70%), likewise does not imply any degradation or transformation. The differences between our findings and those reported elsewhere may be attributed to several factors. First, the chemical composition of *M. communis* EO is known to vary widely according to geographical origin, edaphoclimatic conditions, harvest season, plant organ, and chemotype. Previous studies have identified both 1,8-cineole-rich and linalool/α-pinene-rich chemotypes, with considerable quantitative variability among them [14]. In our study, the plant material was collected from a specific microclimate and altitude, during a particular phenological stage, which may favor increased biosynthesis of eucalyptol. Trans-verbenol (5.65%), which appeared exclusively in the blend, may reflect mild oxidative processes involving α-pinene or related monoterpenes during extraction or storage [15]; however, since no time-course experiments were performed, this remains speculative and is not interpreted as a confirmed transformation.

Hydrocarbon monoterpenes such as α-thujene (1.61%) and α-pinene (6.83%) persisted across samples due to their volatility and chemical stability, while oxygenated sesquiterpenes such as caryophyllene oxide remained minimal (<1%), consistent with their lower abundance and susceptibility to thermal effects. These patterns align with prior studies reporting that blending essential oils modifies their apparent composition and can influence their biological activity through interactions among phenolic, alcoholic, and hydrocarbon constituents [16].

Despite these analytical considerations, the biological implications remain clear. The antimicrobial activity of essential oils is strongly linked to their molecular structure. Compounds such as carvacrol and thymol are known to increase microbial membrane permeability, leading to leakage of intracellular contents and cell death [17]. Essential oils can also inhibit biofilm formation, modulate extracellular polymeric substance (EPS) production, and interfere with mitochondrial respiration, thereby suppressing fungal metabolism and growth [18,19]. In the Oregano/Myrtle blend, carvacrol likely serves as the principal antifungal agent, with γ-terpinene and p-cymene, and other minor constituents may enhance membrane-penetration and synergistically potentiate bioactivity. These interactions help explain the superior antifungal performance of blended oils compared to single components.

### 2.2. Effect of Free and Encapsulated EOs Formulation Vapor on Aspergillus flavus

**Figure 1:**
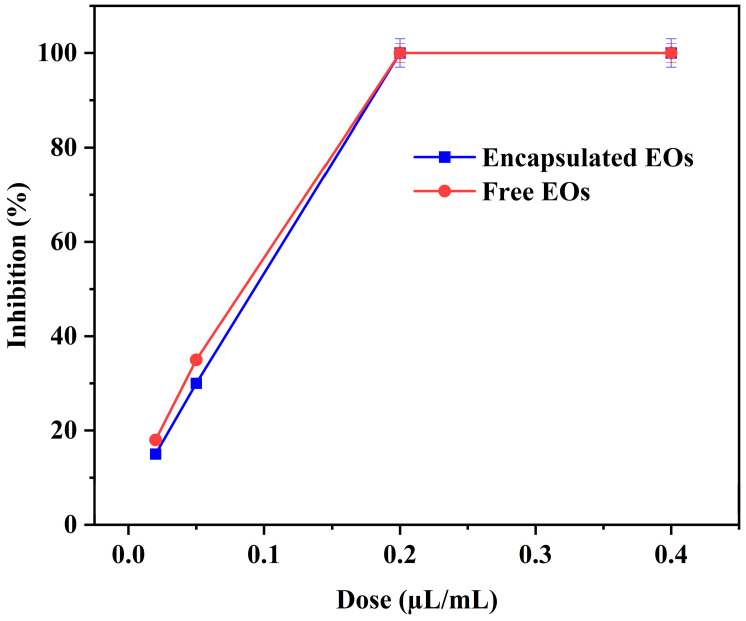
Dose-dependent antifungal activity of free and encapsulated EO vapors against *A. flavus*. Values are mean ± SD (n = 3). Differences were considered significant at *p* ≤ 0.05.

The antifungal efficacy of the essential oil (EO) vapors exhibited a clear dose-dependent increase in inhibitory activity. As shown in Figure 1, comparison of the antifungal effects of free and microencapsulated EOs against *A. flavus* revealed no statistically significant differences between the two formulations at equivalent concentrations (*p* > 0.05). At the lowest tested dose (0.03 µL/mL), both formulations showed similar inhibition levels, with free EOs reaching (15.45 ± 0.50%) and encapsulated EOs reaching (15.00 ± 1.00%). A moderate increase in inhibition was observed at 0.06 µL/mL, where free EOs (31.81 ± 1.90%) inhibition compared with (29.96 ± 0.50%) for the encapsulated form (*p* > 0.05). At concentrations of 0.2 µL/mL and above, complete inhibition of fungal growth was achieved for both formulations (*p* ≤ 0.05 compared with lower doses), confirming the strong antifungal potential of the EO vapors at higher concentrations.

These results demonstrate that the encapsulation process did not reduce the antifungal activity of the EOs. In addition, the 5% gelatin–gum Arabic (G-GA) solution used as a control showed no measurable inhibitory activity against *A. flavus* (*p* > 0.05), confirming that the observed antifungal activity originated exclusively from the essential oils. Overall, these findings are consistent with previous reports showing that microencapsulation preserves the antimicrobial activity of essential oils such as lemongrass, mustard, clove, thyme, and cinnamon [20,21].

### 2.3. Effect of Encapsulated EO Formulation on Fungal Contamination

**Figure 2:**
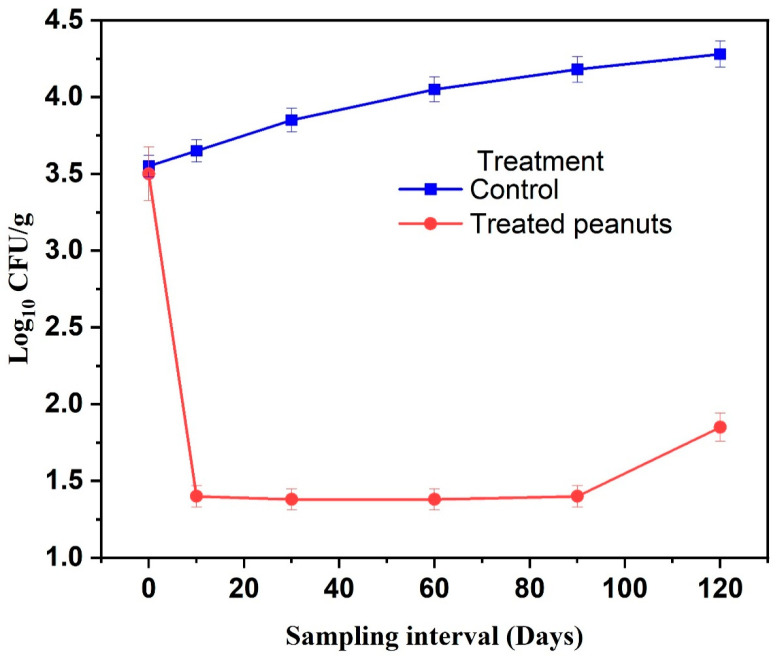
Fungal load in treated and untreated peanuts over time. Values are mean ± SD (n = 3). Differences were considered significant at *p* ≤ 0.05.

The evolution of fungal load in treated and untreated peanuts was monitored throughout storage, revealing statistically significant differences between the two groups (*p* ≤ 0.05) (Figure 2 and Table 2). At the start of the experiment, both control and treated samples exhibited a similar fungal load of (3.52 log_10_ CFU/g; *p* > 0.05). Immediately after treatment, the fungal load in treated peanuts decreased sharply to 1.48 log_10_ CFU/g, representing a reduction of approximately 58% (*p* ≤ 0.05). By day 30, the fungal load stabilized at 1.42 log_10_ CFU/g, corresponding to an overall reduction of about 60% relative to the initial level (*p* ≤ 0.05). This low fungal load remained nearly constant through day 90 (1.43 log_10_ CFU/g; *p* > 0.05). In contrast, untreated peanuts showed a continuous and statistically significant increase in fungal contamination, reaching 4.25 log_10_ CFU/g by day 120 (*p* ≤ 0.05). These results demonstrate that the encapsulated EO formulation effectively suppressed fungal growth for up to 90 days, maintaining microbial stability, whereas untreated peanuts experienced progressive fungal proliferation over time.

Several fungal genera were isolated from the peanuts, including *A. flavus*, *Aspergillus* section *Nigri*, *Penicillium* spp., *Rhizopus*, *Fusarium* sp., and *Alternaria* sp. (Table 2). At day 0, both groups exhibited identical fungal counts (*p* > 0.05). By day 30, statistically significant reductions were observed in treated samples relative to the initial values and to the controls: *A. flavus* (−75%), *A.* section *Nigri* (−70%), *Penicillium* spp. (−68.8%), *Rhizopus* (−75%), *Fusarium* sp. (−75%), and *Alternaria* sp. (−83.3%) (*p* ≤ 0.05). Conversely, untreated peanuts showed a progressive and significant increase in fungal populations.

These results confirm that the microencapsulated EO formulation effectively inhibited the proliferation of major storage fungi in peanuts (*p* ≤ 0.05). The antifungal performance observed is consistent with previous studies using EO microcapsules, including *Lippia turbinata* and *Peumus boldus*, which reported fungal reductions of 31–77% within comparable storage periods [6]. Collectively, these findings demonstrate that microencapsulation not only preserves EO bioactivity but also enables prolonged and statistically validated protection against storage fungi, making the oregano–myrtle formulation a promising alternative to synthetic fungicides for peanut preservation.

### 2.4. Effect on Germination Power

The germination power of both treated and untreated peanuts remained relatively stable throughout the 120-day storage period, as shown in Table 3. Initially, both groups exhibited a germination rate of 52%. By day 120, the control group maintained a germination power of 50.5%, while the treated group showed a slight decrease to 50.1%. This negligible difference indicates that the encapsulated EO formulation had no significant adverse effect on seed viability, preserving germination potential comparable to that of untreated seeds.

In contrast to previous studies that reported noticeable declines in germination following treatment with microencapsulated essential oils, such as *Peumus boldus* (boldo) and *Lippia turbinata* (poleo) formulations, which led to reduced seed viability after extended storage [6], the oregano-myrtle EO blend used in this study effectively maintained seed germination capacity. The stability of germination observed in both control and treated groups may also reflect the favorable storage conditions applied, which helped preserve seed vigor over time.

Overall, these findings demonstrate that the microencapsulated oregano-myrtle EO formulation provides sustained antifungal protection without compromising the germination power of peanut seeds, confirming its suitability for dual applications in both food preservation and agricultural seed storage.

### 2.5. Antioxidant Activity of Free and Encapsulated Essential Oils

**Figure 3:**
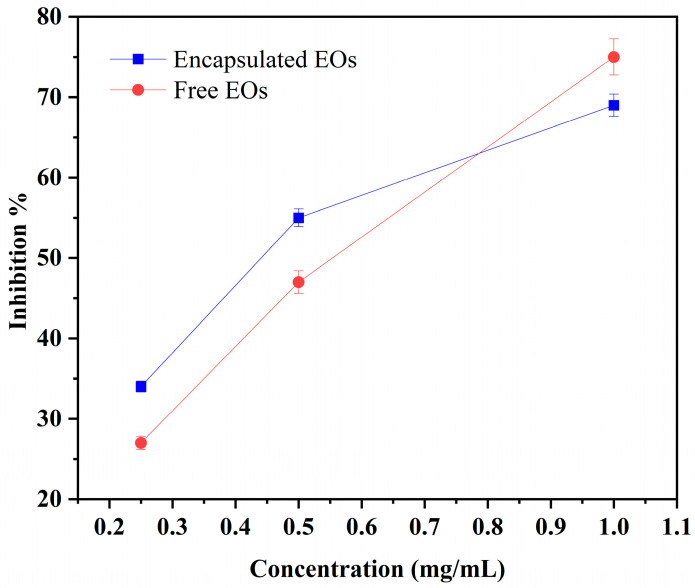
DPPH radical scavenging activity of free and encapsulated essential oils at different concentrations. Values are mean ± SD (n = 3). Differences were considered significant at *p* ≤ 0.05.

The antioxidant activity of free and encapsulated essential oils was evaluated using the DPPH radical scavenging assay at three concentrations (1, 0.5, and 0.25 mg/mL), as shown in Figure 3. Both forms demonstrated concentration-dependent scavenging activity. At the highest concentration (1 mg/mL), free EOs exhibited slightly higher inhibition (75%) compared to encapsulated EOs (71%). However, at lower concentrations, the encapsulated EOs showed comparable or slightly greater inhibition (54% vs. 47% at 0.5 mg/mL, and 34% vs. 27% at 0.25 mg/mL).

The regression equations (y = 62.857x + 13 for free EOs and y = 47.143x + 25.5 for encapsulated EOs) and corresponding IC_50_ values (0.58 mg/mL for free EOs and 0.52 mg/mL for encapsulated EOs) indicate similar antioxidant potency between both forms. These findings confirm that microencapsulation did not significantly compromise the antioxidant capacity of the essential oils, highlighting the technique’s effectiveness in preserving EO bioactivity.

The present results are consistent with previous studies that have reported comparable antioxidant capacities between free and encapsulated essential oils. For instance, Granados et al. [22] demonstrated that microencapsulated *Ocimum gratissimum* L. EO maintained strong antioxidant activity, similar to the free oil, as determined by DPPH and ABTS assays. Likewise, de Oliveira Alencar et al. [23] reported that spray-dried *Cymbopogon citratus* EO retained its radical scavenging capacity with minimal loss of activity after encapsulation. Collectively, these findings reinforce the notion that microencapsulation is a reliable strategy for stabilizing volatile and oxidation-sensitive bioactive compounds while maintaining their functional properties.

## 3. Materials and Methods

### 3.1. Collection and Characterization of Essential Oils

The leaves of *O. compactum* and *M. communis* were harvested after flowering from the region of Moulay Abdessalam, Jbel El Alam, in the province of Larache, northern Morocco. The collected plant materials were air-dried for two weeks in the shade at ambient temperature. Botanical identification was carried out by Dr. Mohammed Chabbi at the Laboratory of Physical Chemistry of Materials, Natural Substances and Environment, Abdelmalek Essaadi University, Tangier, Morocco. Voucher specimens were deposited at the Herbarium of Abdelmalek Essaadi University (TAU), Tangier, under the following accession numbers: *O. compactum* (TAU-OC-2020–001) and *M. communis* (TAU-MC-2020–004). EOs from *O. compactum* and *M. communis* leaves were extracted by hydrodistillation for 3 h using a Clevenger-type apparatus (Kyoto, Japan) and analyzed by gas chromatography-mass spectrometry (GC-MS) as described by Dahmane et al. [24]. The chemical composition of the EO mixture (75:25, *w*/*w*) was also determined using GC-MS following the method of Maliki et al. [25]. Gas chromatographic analyses were conducted using a Shimadzu gas chromatography system (Kyoto, Japan) equipped with a QP2010 mass spectrometer and fitted with an Rtx-5MS fused-silica capillary column (5% diphenyl–95% dimethylpolysiloxane; 30 m × 0.25 mm internal diameter × 0.32 µm film thickness). Helium (99.9% purity) was employed as the carrier gas at a constant flow rate of 1 mL min^−1^. The oven temperature program was initially maintained at 50 °C for 1 min, then increased to 250 °C at a heating rate of 10 °C min^−1^. The mass spectrometer operated in electron impact (EI) mode at 70 eV, with ion scanning conducted over the *m*/*z* range of 40–300. For each analysis, 1 µL of sample was injected in split mode (1:50). All measurements were carried out in triplicate to ensure analytical reproducibility.

### 3.2. Preparation of Dual-Size Microcapsules

Microcapsules were prepared by complex coacervation using gelatin–gum arabic (G-GA) as the wall material. The coacervation process involved emulsifying EOs in an acidic G-GA solution under continuous stirring, allowing polymer aggregation around oil droplets. The pH was then adjusted to 8.0 ± 0.1 using NaOH, followed by physical cross-linking at low temperature (4 °C) to solidify the capsule walls. The microcapsules were washed with distilled water and stored at −20 °C before lyophilization.

Following optimization in our previous work [4], the final formulation consisted of 70% small capsules and 30% large capsules, with a total loading of 0.96 mg g^−1^ of microcapsules. This design achieved the minimum inhibitory concentration (MIC) required for immediate antifungal activity against toxigenic *A. flavus* and other fungal species, while maintaining a sustained vapor-phase release under storage conditions.

Encapsulation efficiency was determined by dissolving 0.5 g of microcapsules in 10 mL of chloroform, shaking for 5 min, and filtering through Whatman No. 1 paper (Merck KGaA, Darmstadt, Germany). The absorbance of the chloroform extract was measured at 220 nm using a UV-visible spectrophotometer (chloroform blank). A calibration curve was prepared from EO mixtures of known concentrations, and encapsulation efficiency was calculated as the percentage of EOs encapsulated relative to the initial amount. All measurements were performed in triplicate.

### 3.3. Effect of Encapsulated EO Vapor on Aspergillus flavus

A toxigenic *A. flavus* strain (RP-6), previously isolated from raw peanuts and characterized for aflatoxin B_1_ (AFB_1_, 2175 µg mL^−1^) and cyclopiazonic acid (CPA, 405.9 µg mL^−1^) production [4], was used. The antifungal activity of the *oregano-myrtle* EO mixture was evaluated using a modified vapor-phase assay according to Soliman et al. [26]. Petri dishes (90 mm diameter) were filled with 15 mL of potato dextrose agar (PDA). Sterilized filter paper disks (30 mm diameter) were fixed to the upper lid were used as the vapor-phase support for free EOs, while 1 mL of solidified sterile agar fixed at the center of the lid was used as the support for microencapsulated EOs. Plates were inoculated with 100 µL of a 10^6^ spores mL^−1^ fungal suspension prepared from a 7-day-old culture. Tested EO concentrations were 0.03, 0.06, 0.2, and 0.4 µL mL^−1^ for both free and encapsulated formulations. Microcapsules contained equivalent EO quantities. The loaded filter paper or microcapsule layer was positioned approximately 6 mm above the agar surface. Plates were sealed with adhesive film and incubated at 27 ± 2 °C for 5 days. Growth inhibition was determined by measuring colony diameters, and inhibition percentages were calculated using standard formulas. All assays were performed in triplicate.

### 3.4. Antioxidant Activity of Free and Encapsulated EOs

The antioxidant activity of the oregano-myrtle EO blend (75:25, *w*/*w*), in both free and encapsulated forms, was evaluated using the DPPH radical scavenging assay according to Haddou et al. [27], with slight modifications. Free EOs were dissolved in methanol, while encapsulated EOs were dispersed in methanol and centrifuged to remove undissolved particles. Aliquots (50 µL) of each sample were mixed with 5 mL of 0.004% methanolic DPPH solution. The mixtures were incubated in the dark at room temperature for 30 min, and absorbance was measured at 517 nm using a spectrophotometer (methanol blank).

The DPPH scavenging activity (%) was calculated using Equation (1):

(1) $$Scavenging\;Activity\;\%=\frac{A_{Control}-A_{sample}}{A_{Control}}\times 100$$

where $A_{\mathrm{control}\;}$ is the absorbance of the DPPH solution without EOs, and $A_{\mathrm{sample}}$ is the absorbance of the EO-DPPH mixture.

### 3.5. Microcosm Assay

Shelled peanuts intended for seed purposes were used. A total of 225 g of peanut kernels were placed in 500 mL plastic flasks. EO formulations were applied at 0.2 mg g^−1^ using a total of 0.96 mg g^−1^ of microcapsules and mixed thoroughly. Untreated kernels served as controls. All flasks were incubated at 20–25 °C for 120 days, and analyses were performed in triplicate at 0, 30, 60, 90, and 120 days.

#### 3.5.1. Evaluation of Fungal Population

For fungal enumeration, 5 g of kernels from each biological replicate were milled and shaken for 30 min with 45 mL of 1 g L^−1^ peptone solution. Serial decimal dilutions up to 10^−3^ were prepared, plated on PDA supplemented with chloramphenicol, and incubated at 25 ± 1 °C for 5–7 days. Results were expressed as CFU g^−1,^ and fungal genera were identified as described in [4].

#### 3.5.2. Evaluation of Germination Power

Germination was assessed using the wet paper method [28]. Ten seeds were incubated at 25 °C for 5 days. Each analysis was performed in triplicate. Germination power was expressed as

(2) $$GP\left(\%\right)=\frac{Number\;of\;germinated\;seeds}{Total\;number\;of\;seeds\;tested}\times 100$$

## 4. Conclusions

This study demonstrates that dual-size gelatin–gum arabic microencapsulation of oregano-myrtle essential oils provides efficient and sustained vapor-phase antifungal protection of stored peanuts. The optimized formulation exhibited a high encapsulation efficiency (83.56%) and ensured both immediate and prolonged release, achieving complete inhibition of *A. flavus* in vitro at concentrations ≥ 0.2 µL/mL and reducing the total fungal load by approximately 60% within the first day of storage, with the suppressive effect maintained for up to 90 days. The encapsulating matrix itself showed no intrinsic antifungal activity, confirming that the bioactivity originated exclusively from the essential oils. Moreover, microencapsulation preserved antioxidant capacity of the oils, with DPPH IC_50_ values comparable to those of free EOs, and maintained peanut germination power at levels equivalent to untreated controls throughout 120 days of storage, demonstrating full compatibility with seed viability. Overall, vapor-active dual-size microcapsules of oregano–myrtle essential oils represent a promising, scalable, and residue-free strategy for controlling peanut mycoflora and associated mycotoxin risks while preserving planting quality. Future work should focus on optimizing the dosage flavor balance, quantifying long-term mycotoxin reduction under extended storage, evaluating efficacy across different peanut varieties and storage environments, and validating the performance and economic feasibility of this technology under real commercial storage conditions.

## Figures and Tables

**Table 1:** Chemical composition of essential oils of *O. compactum*, *M. communis*, and *O. compactum*/*Myrtus communis* at a ratio of 75:25, determined by GC-MS analysis. Values are mean ± SD (n = 3).

N°	Compounds	Essential Oil			
		RT	*Origanum compactum*/*Myrtus communis* (75:25)	*Origanum compactum*	*Myrtus* *communis*
			Area %		
**1**	α-Thujene	5.063	1.61	4.81	-
**2**	α-Pinene	5.192	8.63	3.45	5.20
**3**	Camphene	5.454	-	0.62	-
**4**	β-Thujene	5.549	-	-	0.12
**5**	1-Octen-3-ol	5.959	0.65	2.58	-
**6**	3-Octanone	6.024	0.77	2.11	
**7**	Myrcene	6.092	1.65	4.67	
**8**	Isoamyl isovalerate	6.275	-	-	0.20
**9**	6-Methyl-3-heptanol	6.221	-	0.72	-
**10**	α-Phellandrene	6.368	-	0.99	-
**11**	(+)-4-Carene	6.567	1.91	6.32	-
**12**	m-Cymene	6.715	13.40	9.90	-
**13**	Eucalyptol	6.834	-	-	65.70
**14**	γ-Terpinene	7.275	17.41	28.35	-
**15**	Sabinenehydrate	7.476	-	0.98	-
**16**	Linalool oxide	7.527	-	-	0.24
**17**	α-Terpinolen	7.770	-	0.66	-
**18**	Linalool	7.965	1.85	6.30	2.70
**19**	cis-Verbenol	8.178	-	-	0.53
**20**	α-Campholenal	8.437	-	-	0.41
**21**	Limonene oxide, trans	8.617	-	-	0.15
**22**	Borneol	9.172	-	1.05	-
**23**	Terpinen-4-ol	9.303	0.80	2.82	-
**24**	α-Terpineol	9.532	1.37	-	3.15
**25**	p-menth-1-en-8-ol	9.536	-	2.35	-
**26**	Dihydrocarvone	9.594	-	0.77	-
**27**	Myrtenal	9.605	-	-	0.99
**28**	Verbenone	9.828	-	-	0.73
**29**	2-Isopropyl-1-methoxy-4-methylbenzene	10.252	0.76	-	-
**30**	m-Cresol, 6-tert-butyl-	10.253	-	2.92	-
**31**	Pulegone	10.267	-	-	0.23
**32**	Carvoxime	10.342	-	-	0.74
**33**	Linalyl Acetate	10.341	0.41	-	-
**34**	Thymol	11.030	-	2.54	-
**35**	trans-Pinocarvyl acetate	11.125	-	-	0.19
**36**	o-Mentha-1(7),8-dien-3-ol	11.276	-	-	0.77
**37**	o-Cymen-5-ol	11.290	-	12.02	-
**38**	Carvacrol	11.305	33.83	3.07	-
**39**	trans-Verbenol	11.491	5.65	-	-
**40**	Myrtenyl acetate	11.514	-	-	9.62
**41**	Geraniol acetate	12.216	0.31	-	-
**42**	Methyleugenol	12.607	0.17	-	1
**43**	Caryophyllene	12.952	1.39	-	-
**44**	Caryophyllene oxide	15.194	0.61	-	0.19
	Total identified (%)		92.41	88.42	95.14

**Table 2:** Temporal evolution of fungal species in treated and control peanut samples during storage (log_10_ CFU g^−1^).

Sampling Interval (Days)	Treatment	*Aspergillus* Section *Flavi*	*Aspergillus* Section *Nigri*	*Penicillium* spp.	*Rhizopus*	*Fusarium* sp.	*Alternaria* sp.
**0**	Treated	2 ± 0.1 ^a^	1 ± 0.4 ^a^	0.8 ± 0.7 ^b^	0.4 ± 0.1 ^a^	0.2 ± 0.1 ^a^	0.12 ± 0.1 ^a^
**0**	Control	2 ± 0.1 ^a^	1 ± 0.4 ^a^	0.8 ± 0.7 ^b^	0.4 ± 0.1 ^a^	0.2 ± 0.1 ^a^	0.12 ± 0.1 ^a^
**1**	Treated	0.6 ± 0.5 ^a^	0.4 ± 0.2 ^a^	0.3 ± 0.2 ^a^	0.1 ± 0.9 ^b^	0.05 ± 0.2 ^a^	0.03 ± 0.2 ^a^
**1**	Control	2.1 ± 0.2 ^a^	1.2 ± 0.1 ^a^	0.9 ± 0.3 ^a^	0.5 ± 0.7 ^b^	0.3 ± 0.3 ^a^	0.15 ± 0.1 ^a^
**5**	Treated	0.5 ± 0.1 ^a^	0.35 ± 0.2 ^a^	0.3 ± 0.2 ^a^	0.1 ± 0.3 ^a^	0.05 ± 0.2 ^a^	0.03 ± 0.7 ^b^
**5**	Control	2.2 ± 0.3 ^a^	1.3 ± 0.1 ^a^	1 ± 0.1 ^a^	0.6 ± 0.8 ^b^	0.4 ± 0.1 ^a^	0.2 ± 0.6 ^b^
**30**	Treated	0.5 ± 0.1 ^a^	0.3 ± 0.7 ^b^	0.25 ± 0.5 ^b^	0.1 ± 0.3 ^a^	0.05 ± 0.3 ^a^	0.02 ± 0.5 ^b^
**30**	Control	2.5 ± 0.1 ^a^	1.5 ± 0.7 ^b^	1.2 ± 0.7 ^b^	0.7 ± 0.7 ^b^	0.5 ± 0.3 ^a^	0.25 ± 0.3 ^a^

Mean ± SD (n = 3) standard deviation values in the same column with different upper script letters (a, b) are significantly different (*p* ≤ 0.05).

**Table 3:** Effect of EOs encapsulated formulation on germination power of peanuts over time.

Assays	T0	T1 (30 d)	T2 (60 d)	T3 (90 d)	T4 (120 d)
Control (%)	52 ± 0.9 ^a^	50.5 ± 2.1 ^c^	50.8 ± 1.1 ^b^	50.2 ± 0.9 ^a^	50.5 ± 2.5 ^c^
Treated Peanuts (%)	52 ± 1.2 ^b^	50.2 ± 1.5 ^b^	50.5 ± 3.1 ^c^	49 ± 0.7 ^a^	50.1 ± 2.2 ^c^

Mean ± SD (n = 3) standard deviation values in the same column with different upperscript letters (a–c) are significantly different (*p* ≤ 0.05).

## Data Availability

The original contributions presented in this study are included in the article. Further inquiries can be directed to the corresponding authors.
